# Associations between symptom-based long COVID clusters and long-term quality of life, work and daily activities among individuals testing positive for SARS-CoV-2 at a national retail pharmacy

**DOI:** 10.1186/s41687-024-00797-7

**Published:** 2024-10-22

**Authors:** Manuela Di Fusco, Joseph C. Cappelleri, Alon Yehoshua, Kelly J. Thomas Craig, Mary B. Alvarez, Kristen E. Allen, Thomas M. Porter, Santiago M.C. Lopez, Laura Puzniak, Xiaowu Sun

**Affiliations:** 1 Pfizer Inc, New York, NY USA; 2 CVS Health, Woonsocket, RI USA

**Keywords:** SARS-CoV-2, COVID-19, BNT162b2, long-COVID-19 symptoms, Humanistic, HRQoL, Quality of life, WPAI

## Abstract

**Background:**

Evidence on long COVID symptom clustering patterns among patients with COVID-19 is limited. We summarized long COVID symptoms in clusters defined by number of symptoms co-occurring together, and we assessed Health-Related Quality of Life (HQRoL), Work Productivity and Activity Impairment (WPAI) outcomes across these clusters over time. We assessed associations between the clusters and BNT162b2 vaccination status.

**Methods:**

A prospective longitudinal patient-reported outcomes (PRO) study recruited laboratory-confirmed symptomatic COVID-19 patients seeking testing from a national retail pharmacy. Long COVID-19 symptoms were self-reported by participants at 4-week, 3-month and 6-month surveys. Patient classes identified via latent class analysis (LCA) with long COVID-19 symptoms were simplified into clusters based on number of symptoms. HRQoL and WPAI outcomes were collected using EQ-ED-5L and WPAI: GH questionnaires. Mixed models for repeated measures analyses were conducted to examine associations between exposure groups and outcomes.

**Results:**

The study included 328 participants that were segmented into three groups of long COVID-19 symptoms based on LCA and then simplified by the number of symptoms (Cluster 1 low, <2; Cluster 2 moderate, 2–6; and Cluster 3 high, >6 symptoms). The number of long COVID-19 symptoms was negatively associated with HRQoL and WPAI, whereby participants with high symptom burden (>6 symptoms) had the lowest HRQoL and WPAI scores assessed by absenteeism, presenteeism, work productivity loss, activity impairment, and hours worked metrics. Compared with those unvaccinated and not up-to-date with COVID-19 vaccination, subjects boosted with BNT162b2 consistently reported less symptom burden during the follow-up, regardless of their symptom-based cluster.

**Conclusion:**

Three distinct patient clusters based on frequency of long COVID symptoms experienced different HRQoL and WPAI outcomes over 6 months. The cluster with more concomitant symptoms experienced greater burden than the others. Participants up-to-date with BNT162b2 reported lower symptom burden across all clusters and timeframes.

**Clinical trial registration:**

Clinicaltrials.gov NCT05160636.

**Supplementary Information:**

The online version contains supplementary material available at 10.1186/s41687-024-00797-7.

## Introduction

A growing number of studies show that patients with COVID-19 can experience symptoms during both the acute and the long-term phase of the infection. While no agreed upon definition exists yet, the long-term phase, known as ‘long COVID’, is characterized by prolonged or emerging symptoms and health conditions [1–5].

Research shows that both acute and long COVID symptoms can negatively impact wellbeing and activity, leading to profound societal and economic burden [2–7]. Indeed, our prior study leveraged the CDC symptoms list and found that symptomatic participants testing at a US retail pharmacy experienced quality of life detriments, activity and work impairments over 6 months following infection [7].

Systematic literature reviews suggest that COVID-19 vaccination may have protective effects against acute and long COVID [8–10], commonly reported as the reduction in the incidence of symptoms. In our prior study, we found that participants boosted with BNT162b2 before a breakthrough infection were associated with significantly lower odds of long COVID (defined as ≥2 or 3 symptoms at Week 4) compared with those primed (who completed the primary series) or unvaccinated [7].

Identifying clusters of COVID-19 patients can improve the characterization of the disease and can allow capturing potential variations in vaccine performance. However, to date, studies reporting COVID-19 patient clusters remain rare and mainly focused on symptom types [3, 10–14]. A Latent Class Analyses applied to long COVID symptoms in our study found that phenotypes differ by prevalence rather than by type of symptoms [15]. A clustering approach based on the frequency of symptoms has not been applied yet and could supplement existing efforts aimed at understanding underlying patterns and variations in COVID-19 symptomatology, vaccine performance, and health impacts.

As such, we conducted follow-up analyses to our prior study [7] with the aims to (1) identify patient clusters based on number of symptoms, (2) assess associations between these patient clusters and HRQoL, activity and work-related outcomes over time, and (3) determine associations between BNT162b2 and long COVID symptoms across these patient clusters, over time.

## Methods

### Study design and participants

This prospective patient-reported outcomes (PRO) study design has been previously described (clinicaltrials.gov NCT05160636) [16]. The study targeted consented adult outpatients who tested positive for COVID-19 using reverse transcription-polymerase chain reaction (RT-PCR) at a CVS Health^®^ test site within the United States (US) and self-reported at least one symptom upon testing. The recruitment was conducted online; it spanned from January 31, 2022, through April 30, 2022, with follow-up completed before October 31, 2022. The participants eligible for these analyses were those who self-reported persistent long COVID symptoms at week 4 post-infection, the start of ‘long COVID’, per CDC definition [1].

### Baseline characteristics

Baseline characteristics were obtained via the CVS Health pre-test screening questionnaire, which included self-reported demographics, comorbidities, and COVID-19 vaccination history.

### Exposure groups

The analyses included two main exposure groups of interest.

The first group included patient clusters defined according to the number of COVID-19 symptoms co-occurring together. The methods used to determine the exposure distributions by cluster are described in the Statistical Methods.

The second group was defined according to vaccination status. In this group, participants were categorized into three mutually exclusive groups according to their self-reported vaccination status: (1) “unvaccinated” if they did not report COVID-19 vaccination history prior to testing; (2) “boosted,” if they received at least one dose after BNT162b2 primary series; or (3) “primed”, if they received BNT162b2 primary series. BNT162b2 was the most used vaccine type used in the US during the study period [17].

### Outcomes and data sources

The outcomes of interest for these analyses included long COVID-19 symptoms, HRQoL, work productivity and activity impairment. The outcomes were collected longitudinally via online questionnaires that subjects in the study were asked to complete through six months following enrollment [16]. The long COVID symptoms were collected via an online long COVID symptom questionnaire. As previously described, the questionnaire was comprised of a list of 20 symptoms based on the CDC list [1] The questionnaire was completed at 4-week, 3-month and 6-month following enrollment.

Validated PRO instruments were administered at 4-week, 3-month and 6-month post-enrollment to assess HRQoL using the EQ-5D-5L questionnaire [18] and to evaluate paid and unpaid work losses using the Work Productivity and Activity Impairment questionnaire (WPAI; General Health v2.0 measure) [19, 20] At each time point, five dimensions of the EQ-5D-5L were summarized using US-based weights (per Pickard et al. [21]) as the Utility Index (UI). The HRQoL tool also uses a visual analogue scale (VAS). For EQ-5D-5L interpretation, lower scores for both UI and VAS indicate a decreased self-reported overall HRQoL. For WPAI interpretation, higher scores indicate a greater self-reported activity impairment and work productivity loss.

### Data analyses and bias

Analyses were conducted to (1) identify patient clusters based on number of symptoms (further described in Statistical Methods), (2) evaluate associations between the identified patient clusters and HRQoL, activity and work-related outcomes, over time, and (3) evaluate associations between BNT162b2 and long COVID symptoms across the identified patient clusters, over time.

In an attempt to minimize data missingness, loss of data, sample reduction, selection bias, and other bias due to non-participation and loss to follow-up, participants were compensated after completing each questionnaire, received reminders, and were not allowed to skip surveys. Any symptoms not reported by patients in the questionnaires were considered absent and all the available symptom data were included in the analysis. When scoring the EQ-5D-5L UI and WPAI, no adjustment was made for missing data, per guidelines, and all the available data was used [20, 21]. Finally, to help minimize misclassification and information bias, the same instruments measuring the outcomes of interest were applied to all the exposure groups.

### Statistical methods

Categorical variables including number-based category were summarized with count and frequency. Continuous variables including the EQ-5D-5L and WPAI questionnaire scores were summarized with mean and standard deviation.

Latent class analysis (LCA) was used to identify patient clusters with specific patterns of long COVID symptoms. Patient clusters were determined by taking into consideration smaller Bayesian information criterion, no small class sizes, and clinical interpretation [22]. Clusters based on number of symptoms were determined via the cross-tabulation with previously described patient classes identified via LCA [15]. The agreement of number of symptoms-based clusters and LCA identified classes was assessed by using weighted kappa coefficient. A kappa coefficient > 0.75 indicates excellent agreement. Chi-square tests were used to compare the distribution of number-based category of boosted subjects with those of primed subjects or unvaccinated subjects.

To investigate the relationship between PRO scores and the category of number of COVID-19 related symptoms, mixed-model for repeated measures analyses were conducted with score as dependent variable, and assessment time (4-week, 3-month, and 6-month), category of number of COVID-19-related symptoms, and their interaction as covariates [23]. The model controlled for a variety of covariates and used an unstructured covariance matrix for categorical assessment time [16]. The variables that the model controlled for were pre-COVID-19 score, index vaccination status and its interaction with time, age, gender, race/ethnicity, region, social vulnerability index category, number of acute respiratory infection symptoms on index day, previously tested positive, high-risk settings, and immune-compromised conditions. Scores from EQ-5D-5L (VAS and UI), and WPAI questionnaires were analyzed using separate models.

To investigate the relationship between the category of number of COVID-19 related symptoms and vaccination status, an ordinal logistic model [24] for category of number of COVID-19 related symptoms was fit with assessment time, vaccination status and their interaction controlling for covariates using generalized estimation equation (GEE) approach. Missing data at each timepoint were not imputed. All available data at each time point were included in the analysis. Tukey’s adjustment was conducted for the comparisons of least-square means between study cohorts at each time point.

Analyses were performed with SAS Version 9.4 (SAS Institute, Cary, NC) with significance testing (two-sided) at the 0.05 level, with no adjustment for multiplicity. The study followed the Strengthening the Reporting of Observational Studies in Epidemiology (STROBE) reporting guideline [25] (Appendix).

## Results

Of the 39,889 eligible individuals outreached, a final cohort of 328 participants met inclusion criteria for our prior study and the current analyses. The cohort has been described previously [7]. Figure S1 shows the patient flow of the study.

Patient classes identified via latent class analysis (Table S1) [15] were simplified into clusters defined by the number of co-occurring self-reported symptoms: Cluster 1 low, <2; Cluster 2 moderate, 2–6; and Cluster 3 high, >6 symptoms (Table 1). The number of symptom-based clusters had excellent agreement with LCA identified classes across all time points and all time points combined with all weighted kappa coefficients > 0.93 (Table S2).

There were 48.8%, 33.2% and 18.0% of participants, respectively, in Cluster 1, Cluster 2, and Cluster 3. Table 1 reports the baseline characteristics for each cluster. The three clusters were relatively similar, with main differences in the prevalence of underlying comorbidities and pre-infection vaccination status. The composition of the clusters was stable over time (Table S2).

**Table 1:** Participants characteristics by clusters based on number of symptoms at week 4

	Cluster 1: <2 symptoms	Cluster 2: 2–6 symptoms	Cluster 3: >6 symptoms	*P* value^a^
Total, n (%)	160 (48.8%)	109 (33.2%)	59 (18.0%)	
Age, years				
Mean, SD	41.6 (15.0)	42.8 (14.8)	41.5 (12.6)	0.777
Age group				0.548
18–29	36 (22.5%)	26 (23.9%)	11 (18.6%)	
30–49	81 (50.6%)	47 (43.1%)	32 (54.2%)	
50–64	27 (16.9%)	27 (24.8%)	13 (22.0%)	
≥65	16 (10.0%)	9 (8.3%)	3 (5.1%)	
Gender				0.071
Female	111 (69.4%)	81 (74.3%)	50 (84.7%)	
Male	49 (30.6%)	28 (25.7%)	9 (15.3%)	
Race/Ethnicity				0.850
White or Caucasian (not Hispanic or Latino)	112 (70.0%)	78 (71.6%)	44 (74.6%)	
Black or African American	5 (3.1%)	4 (3.7%)	4 (6.8%)	
Hispanic	25 (15.6%)	14 (12.8%)	5 (8.5%)	
Asian	9 (5.6%)	5 (4.6%)	2 (3.4%)	
Other	9 (5.6%)	8 (7.4%)	4 (6.8%)	
US geographic region				0.199
Northeast	21 (13.1%)	18 (16.5%)	2 (3.4%)	
South	90 (56.3%)	63 (57.8%)	35 (59.3%)	
Midwest	29 (18.1%)	18 (16.5%)	16 (27.1%)	
West	20 (12.5%)	10 (9.2%)	6 (10.2%)	
Social vulnerability index, Mean (SD)	0.42 (0.21)	0.44 (0.22)	0.47 (0.22)	0.208
Previously tested positive	58 (36.3%)	38 (34.9%)	25 (42.4%)	0.612
Work in healthcare	17 (10.6%)	14 (12.8%)	6 (10.2%)	0.816
Work in high-risk setting	12 (7.5%)	13 (11.9%)	8 (13.6%)	0.305
Live in high-risk setting	5 (3.1%)	7 (6.4%)	4 (6.8%)	0.354
Self-reported comorbidity				
Number of comorbidities, Mean (SD)	0.23 (0.54)	0.48 (0.70)	0.46 (0.77)	0.003
Asthma or Chronic lung disease	7 (4.4%)	13 (11.9%)	10 (16.9%)	0.008
Cirrhosis of the liver	0 (0.0%)	1 (0.9%)	0 (0.0%)	0.365
Immunocompromised conditions or weakened immune system^b^	7 (4.4%)	7 (6.4%)	2 (3.4%)	0.629
Diabetes	3 (1.9%)	5 (4.6%)	3 (5.1%)	0.344
Heart conditions or hypertension	14 (8.8%)	19 (17.4%)	8 (13.6%)	0.103
Overweight or obesity	5 (3.1%)	7 (6.4%)	4 (6.8%)	0.354
At least 1 comorbidity	28 (17.5%)	39 (35.8%)	20 (33.9%)	0.001
Vaccination status				0.003
Unvaccinated	72 (45.0%)	43 (39.4%)	40 (67.8%)	
Primed	40 (25.0%)	32 (29.4%)	14 (23.7%)	
Boosted	48 (30.0%)	34 (31.2%)	5 (8.5%)	

*Abbreviations SD* standard deviation, *US* United States

^a^*P* value refers to the comparison among categories of <2, 2–6 and >6 symptoms

^b^Immunocompromised conditions includes compromised immune system (such as from immuno-compromising drugs, solid organ or blood stem cell transplant, HIV, or other conditions), conditions that result in a weakened immune system, including cancer treatment, and kidney failure or end stage renal disease

**Fig. 1:**
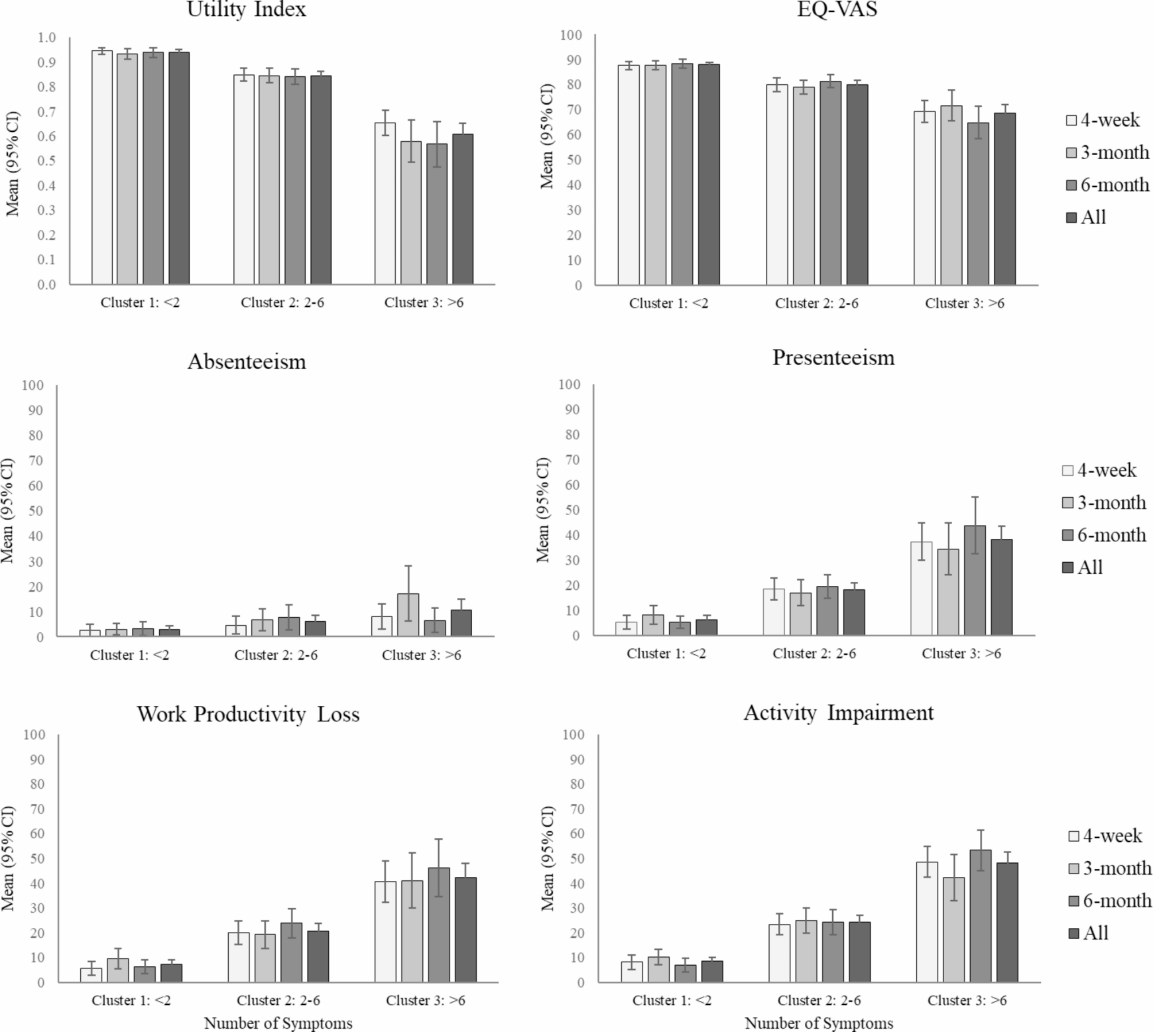
Health-related Quality of Life (HQRoL) and Work Productivity and Activity Impairment (WPAI) outcomes by symptom cluster and time point

Figure 1 shows the HRQoL and WPAI scores at each time point, stratified by the three patient clusters. Both HRQoL and WPAI scores worsened with greater number of symptoms. Cluster 1 (low: <2 symptoms) experienced significantly better HRQoL (EQ-VAS and utility index) than Cluster 3 (high: >6 symptoms) across all time points and overall (Table S3, for all comparisons of Cluster 1 vs. Cluster 3; *P* < 0.001). Observed work-related impairments increased with a greater number of reported COVID-19-related symptoms (Fig. 1). Participants in Cluster 3 reported more presenteeism, work productivity lost and activity impairment when compared to participants in Cluster 1 across all time points and overall (Table S3, all *P* < 0.001), and more absenteeism in general but statistically significant at 4-week, 3-month and overall (*P* = 0.015, 0.001, 0.177, and <0.001 for 4-week, 3-month, 6-month and overall, respectively). Overall, participants in Cluster 3 missed ~4.4-fold as many working hours than those in Cluster 1 (Figure S2; Table S3, *P* = 0.003 overall).

**Fig. 2:**
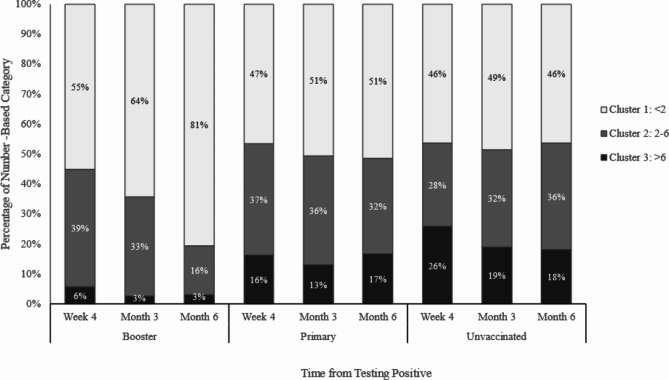
Associations between BNT162b2 vaccination status and symptom-based cluster, by time point

Figure 2 and Table S4 show that participants boosted with BNT162b2 (*N* = 87) had fewer number of COVID-19-related symptoms compared with only the primary vaccination series (primed, *N* = 86) or the unvaccinated (unvaccinated, *N* = 155). The boosted participants reported less symptom burden (i.e., high, >6 symptoms) (4-week: boosted [6%] vs. primed [16%], *P* = 0.027; boosted [6%] vs. unvaccinated [26%], *P* < 0.001; 3-month: boosted [3%] vs. primed [13%], *P* = 0.021; boosted [3%] vs. unvaccinated [19%], *P* = 0.001; 6-month: boosted [3%] vs. primed [17%], *P* = 0.007; boosted [3%] vs. unvaccinated [18%], *P* = 0.003). At month 6, 81% of the boosted participants were in Cluster 1 in comparison with 51% (*P* < 0.001) of the primed and 46% (*P* < 0.001) of the unvaccinated participants. While the distribution of the patient clusters was relatively stable over time for those unvaccinated and primed, a greater proportion of participants boosted with BNT162b2 moved away from high symptom burden to lower symptom burden clusters over time, especially between Month 3 and Month 6. Moreover, a greater proportion of boosted participants reported complete alleviation of symptoms (i.e., 0 symptoms at month 6) compared with those unvaccinated (54% versus 38%, *P* = 0.037).

## Discussion

These analyses identified three patient clusters based on number of COVID-19 symptoms co-occurring together and found that clusters presented differences in HRQoL, work productivity and activity. Both HRQoL and WPAI scores worsened with a greater number of symptoms, across all time points. Cluster 3, self-reporting > 6 long-COVID symptoms, experienced the largest symptom burden, with markedly lower HRQoL and higher WPAI scores than Cluster 1, self-reporting <2 symptoms.

Study participants that were most up-to-date with BNT162b2 vaccination (‘boosted’) were associated with less symptom burden overall and at all time points compared to participants that were unvaccinated or primed.

We have previously reported on the associations between BNT162b2 vaccination status and these humanistic and economic domains [7]. Individuals either fully- (i.e., boosted) or partially vaccinated with BNT162b2 experienced fewer and less durable symptoms than those unvaccinated, which translated to improved HRQoL and lessened work-related impairments [7]. The present study extends upon these findings to further delineate COVID-19 symptomatology by clustering participants by their symptom burden. Strong trends illustrated a direct linear relationship whereby participants with greater number of long COVID symptoms were associated with significantly worse PROs scores for well-being, activity and work-related impairments.

Limitations of this study design have been previously described [7, 16]. The data collected were exclusively self-reported and subject to errors, biases including recall, social desirability, and selection bias due to loss of follow-up (i.e., 21% were lost to follow-up by 6 months). Other considerations for generalizability include overrepresentation of females, exclusion of pediatric and adolescent population and individuals with heterologous vaccination schedules, and some PROs (e.g., WPAI) were limited by small sample size. Antiviral treatment history is not accounted for in this analysis and clinical corroboration of symptoms reported is lacking, along with the assessment of severity of symptoms. The population and their observations of symptom number is not static, as participants may move from one category of symptom burden group over time, if their clinical sequalae changes. As such, another limitation of this study includes the inability to draw direct between-group comparisons.

Despite these limitations, this study and follow-up analyses have notable strengths. The PROs obtained from a national study confer the patients’ perspectives of the sustained impact of SARS-CoV-2 infection. This study utilized innovative methods to capture, analyze, and report patient experiences. An expansive national footprint of ~5000 brick-and-mortar locations was leveraged to provide equitable and validated RT-PCR testing for COVID-19 that enabled recruitment of a diverse study population. This community-based study with digital tools to recruit participants, capture PROs, and manage the study supports health equity to scale research for participants in a non-clinical setting. Finally, these follow-up analyses capture the variability of symptoms and expand the understanding of the manifestation of COVID-19 symptomatology. To date, current studies describing COVID-19 patient clusters remain limited, and mainly focus on long COVID conditions [3, 10–14].

To our knowledge, this is the first application of a clustering approach based on the frequency of concomitant symptoms. The clustering approach suggests that long COVID is a heterogeneous health condition with distinct sub-populations experiencing different long-term outcomes. As such, these analyses provide additional insights on the characterization of COVID-19 symptoms, and supplement efforts aimed at elucidating underlying patterns, trajectories, and variations in COVID-19 symptomatology, as well as vaccine impact on symptoms alleviation. Future studies could expand on the characterization of COVID-19 symptomatology across various strata (e.g. age or pre-existing conditions) and could explore additional clustering criteria (e.g., symptom severity). Such studies could identify additional sub-populations and support tailoring appropriate interventions by subgroup.

## Conclusions

This study identified three patient clusters based on number of COVID-19 symptoms co-occurring together and found that the clusters were associated with differences in HRQoL, activity and work-related outcomes. Participants with higher symptom burden reported higher HRQoL and WPAI loss. In line with the growing evidence illustrating the benefits of vaccination against COVID-19, BNT162b2 vaccination was associated with less symptom burden overall and at all time points, and across clusters. Study participants that were most up to date with BNT162b2 before their breakthrough infection reported greater benefits than those primed or unvaccinated against COVID-19, highlighting the importance of staying up to date with COVID-19 vaccination recommendations. These findings supplement existing studies suggesting that long COVID is a heterogeneous condition with distinct sub-populations experiencing different outcomes and potentially requiring tailored interventions.

## Electronic supplementary material

Below is the link to the electronic supplementary material.

Supplementary Material 1

Supplementary Material 2

## Data Availability

Aggregated data that support the findings of this study are available upon reasonable request from the corresponding author MDF, subject to review. These data are not publicly available due to research participant privacy limitations.

## Appendix

### STROBE Statement—Checklist of items that should be included in reports of *cohort studies*

**Table:**

	Item No	Recommendation	Page No
**Title and abstract**	1	(*a*) Indicate the study’s design with a commonly used term in the title or the abstract	1
		(*b*) Provide in the abstract an informative and balanced summary of what was done and what was found	2–3
**Introduction**			
Background/rationale	2	Explain the scientific background and rationale for the investigation being reported	3–4
Objectives	3	State specific objectives, including any prespecified hypotheses	4
**Methods**			
Study design	4	Present key elements of study design early in the paper	4–5
Setting	5	Describe the setting, locations, and relevant dates, including periods of recruitment, exposure, follow-up, and data collection	4–6
Participants	6	(*a*) Give the eligibility criteria, and the sources and methods of selection of participants. Describe methods of follow-up	4–6
		(*b*) For matched studies, give matching criteria and number of exposed and unexposed	N/A
Variables	7	Clearly define all outcomes, exposures, predictors, potential confounders, and effect modifiers. Give diagnostic criteria, if applicable	5–8
Data sources/measurement	8*	For each variable of interest, give sources of data and details of methods of assessment (measurement). Describe comparability of assessment methods if there is more than one group	5–8
Bias	9	Describe any efforts to address potential sources of bias	6, 8
Study size	10	Explain how the study size was arrived at	4, 8, 22
Quantitative variables	11	Explain how quantitative variables were handled in the analyses. If applicable, describe which groupings were chosen and why	6–8
Statistical methods	12	(*a*) Describe all statistical methods, including those used to control for confounding	6–8
		(*b*) Describe any methods used to examine subgroups and interactions	
		(*c*) Explain how missing data were addressed	
		(*d*) If applicable, explain how loss to follow-up was addressed	
		(*e*) Describe any sensitivity analyses	
**Results**			
Participants	13*	(a) Report numbers of individuals at each stage of study—eg numbers potentially eligible, examined for eligibility, confirmed eligible, included in the study, completing follow-up, and analysed	8, 24
		(b) Give reasons for non-participation at each stage	24
		(c) Consider use of a flow diagram	24
Descriptive data	14*	(a) Give characteristics of study participants (eg demographic, clinical, social) and information on exposures and potential confounders	8–10
		(b) Indicate number of participants with missing data for each variable of interest	N/A
		(c) Summarise follow-up time (eg, average and total amount)	4–5 (pre-defined)
Outcome data	15*	Report numbers of outcome events or summary measures over time	10–12
Main results	16	(*a*) Give unadjusted estimates and, if applicable, confounder-adjusted estimates and their precision (eg, 95% confidence interval). Make clear which confounders were adjusted for and why they were included	7, 22, 23
		(*b*) Report category boundaries when continuous variables were categorized	8
		(*c*) If relevant, consider translating estimates of relative risk into absolute risk for a meaningful time period	N/A
Other analyses	17	Report other analyses done—eg analyses of subgroups and interactions, and sensitivity analyses	25
**Discussion**			
Key results	18	Summarise key results with reference to study objectives	13
Limitations	19	Discuss limitations of the study, taking into account sources of potential bias or imprecision. Discuss both direction and magnitude of any potential bias	14
Interpretation	20	Give a cautious overall interpretation of results considering objectives, limitations, multiplicity of analyses, results from similar studies, and other relevant evidence	13–15
Generalisability	21	Discuss the generalisability (external validity) of the study results	13, 14
**Other information**			
Funding	22	Give the source of funding and the role of the funders for the present study and, if applicable, for the original study on which the present article is based	18

*Give information separately for exposed and unexposed groups

## Abbreviations

BNT162b2: Pfizer-BioNTech COVID-19 vaccine
COVID-19: Coronavirus disease 2019
EQ-5D-5L: EuroQoL Group 5-dimension and 5-level
HRQoL: Health-related quality of life
PRO: Patient-reported outcomes
RT-PCR: Reverse transcription-polymerase chain reaction
SARS-CoV-2: Severe acute respiratory syndrome coronavirus 2
UI: Utility index
VAS: Visual analogue scale
WPAI: Work productivity and activity impairment
