# Supplementary material for: Associations between symptom-based long COVID clusters and long-term quality of life, work and daily activities among individuals testing positive for SARS-CoV-2 at a national retail pharmacy
Source: J Patient Rep Outcomes. 2024 Oct 22;8:122. doi: 10.1186/s41687-024-00797-7 (PMC11496399; doi:10.1186/s41687-024-00797-7)
Supplement: Supplementary file 1 — Supplementary Material 1 [file 41687_2024_797_MOESM1_ESM.docx]

**Supplemental Tables**

**Table S1**. *Results of Latent Class Analysis (LCA; class 1, 2, and 3 by sample size) Segmented by Number of COVID-19-related Symptoms over the 6-month Observation Period*

| No. Symptoms | 4-Week | | | 3-Month | | | 6-Month | | | All Time Points Combined | | |
| --- | --- | --- | --- | --- | --- | --- | --- | --- | --- | --- | --- | --- |
|  | Class 1  (n=165) | Class 2  (n=100) | Class 3  (n=63) | Class 1  (n=154) | Class 2  (n=103) | Class 3  (n=35) | Class 1  (n=149) | Class 2  (n=78) | Class 3  (n=33) | Class 1  (n=468) | Class 2  (n=281) | Class 3  (n=131) |
| 0 | 63.0% |  |  | 81.8% |  |  | 79.9% |  |  | 74.6% |  |  |
| 1 | 32.7% | 2.0% |  | 18.2% | 1.0% |  | 18.8% |  |  | 23.5% | 1.1% |  |
| 2 | 4.2% | 34.0% |  |  | 29.1% |  | 0.7% | 30.8% |  | 1.7% | 31.3% |  |
| 3 |  | 28.0% |  |  | 25.2% |  | 0.7% | 25.6% |  | 0.2% | 26.3% |  |
| 4 |  | 21.0% |  |  | 19.4% |  |  | 16.7% |  |  | 19.2% |  |
| 5 |  | 7.0% | 3.2% |  | 11.7% |  |  | 11.5% |  |  | 10.0% | 1.5% |
| 6 |  | 6.0% | 6.3% |  | 9.7% |  |  | 10.3% | 3.0% |  | 8.5% | 3.8% |
| 7 |  | 2.0% | 14.3% |  | 3.9% | 14.3% |  | 5.1% | 9.1% |  | 3.6% | 13.0% |
| 8 |  |  | 17.5% |  |  | 2.9% |  |  | 18.2% |  |  | 13.7% |
| 9 |  |  | 15.9% |  |  | 28.6% |  |  | 15.2% |  |  | 19.1% |
| 10 |  |  | 17.5% |  |  | 8.6% |  |  | 6.1% |  |  | 12.2% |
| 11 |  |  | 9.5% |  |  | 28.6% |  |  | 9.1% |  |  | 14.5% |
| 12 |  |  | 6.3% |  |  | 2.9% |  |  | 24.2% |  |  | 9.9% |
| 13 |  |  | 1.6% |  |  | 5.7% |  |  | 3.0% |  |  | 3.1% |
| 14 |  |  | 3.2% |  |  | 2.9% |  |  | 6.1% |  |  | 3.8% |
| 15 |  |  | 1.6% |  |  | 5.7% |  |  | 3.0% |  |  | 3.1% |
| 16 |  |  | 3.2% |  |  |  |  |  |  |  |  | 1.5% |
| 17 |  |  |  |  |  |  |  |  | 3.0% |  |  | 0.8% |

Footnotes:

Abbreviations: No, number; SD, standard deviation; n, sample size.

**Table S2**. *Cross-tabulation of Number of Symptoms-based Clusters and LCA Identified Classes*

| LCA identified class | Number of symptoms-based cluster | | | | | | | | | | | | |
| --- | --- | --- | --- | --- | --- | --- | --- | --- | --- | --- | --- | --- | --- |
|  | 4-week | | | 3-month | | | 6-month | | | All | | | |
|  | < 2 | 2 – 6 | > 6 | < 2 | 2 – 6 | > 6 | < 2 | 2 – 6 | > 6 | < 2 | 2-6 | > 6 |  |
| Total | 160 | 109 | 59 | 155 | 98 | 39 | 147 | 77 | 36 | 462 | 284 | 134 |  |
| Total % | 49% | 33% | 18% | 53% | 34% | 13% | 57% | 30% | 14% | 53% | 32% | 15% |  |
| Class1 | 158 | 7 | 0 | 154 | 0 | 0 | 147 | 2 | 0 | 459 | 9 | 0 |  |
| Class2 | 2 | 96 | 2 | 1 | 98 | 4 | 0 | 74 | 4 | 3 | 268 | 10 |  |
| Class3 | 0 | 6 | 57 | 0 | 0 | 35 | 0 | 1 | 32 | 0 | 7 | 124 |  |
| Weighted kappa | 0.935 | | | 0.976 | | | 0.963 | | | 0.956 | | | |

**Table S3**. *Summary of EQ-5D-5L and WPAI Scores*

| Measure | Time | < 2 symptoms | | 2 – 6 symptoms | | > 6 symptoms | | P value *^a^* | | |
| --- | --- | --- | --- | --- | --- | --- | --- | --- | --- | --- |
|  |  | n | Mean (SD) | n | Mean (SD) | n | Mean (SD) | < 2 vs. 2 – 6 | < 2 vs. > 6 | 2 – 6 vs. > 6 |
| EQ-VAS | 4-week | 159 | 87.6 (10.9) | 104 | 80.0 (14.2) | 57 | 69.3 (16.7) | <0.001 | <0.001 | <0.001 |
|  | 3-month | 154 | 87.8 (10.8) | 97 | 78.9 (13.9) | 39 | 71.7 (19.2) | <0.001 | <0.001 | 0.008 |
|  | 6-month | 146 | 88.5 (11.0) | 75 | 81.4 (11.0) | 36 | 64.9 (20.2) | 0.003 | <0.001 | <0.001 |
|  | All | 459 | 87.9 (10.9) | 276 | 80.0 (13.3) | 132 | 68.8 (18.5) | <0.001 | <0.001 | <0.001 |
| Utility Index | 4-week | 160 | 0.944 (0.092) | 109 | 0.849 (0.147) | 59 | 0.654 (0.203) | <0.001 | <0.001 | <0.001 |
|  | 3-month | 155 | 0.934 (0.135) | 98 | 0.845 (0.148) | 39 | 0.579 (0.269) | 0.001 | <0.001 | <0.001 |
|  | 6-month | 147 | 0.938 (0.116) | 77 | 0.840 (0.137) | 36 | 0.567 (0.281) | <0.001 | <0.001 | <0.001 |
|  | All | 462 | 0.939 (0.115) | 284 | 0.845 (0.144) | 134 | 0.609 (0.247) | <0.001 | <0.001 | <0.001 |
| WPAI: GH |  |  |  |  |  |  |  |  |  |  |
| Absenteeism | 4-week | 112 | 2.4 (13.8) | 86 | 4.5 (16.9) | 34 | 8.0 (14.9) | 0.905 | 0.015 | 0.010 |
|  | 3-month | 113 | 2.9 (12.1) | 73 | 6.6 (19.0) | 26 | 17.1 (28.4) | 0.682 | 0.001 | 0.002 |
|  | 6-month | 108 | 3.1 (14.6) | 58 | 7.7 (19.7) | 21 | 6.5 (11.0) | 0.210 | 0.177 | 0.636 |
|  | All | 333 | 2.8 (13.5) | 217 | 6.1 (18.3) | 81 | 10.6 (19.9) | 0.350 | <0.001 | 0.002 |
| Presenteeism | 4-week | 110 | 5.4 (15.0) | 84 | 18.6 (20.3) | 34 | 37.4 (22.2) | <0.001 | <0.001 | <0.001 |
|  | 3-month | 112 | 8.1 (20.2) | 71 | 17.0 (22.1) | 25 | 34.4 (26.3) | 0.077 | <0.001 | 0.013 |
|  | 6-month | 106 | 5.3 (13.1) | 57 | 19.5 (18.5) | 21 | 43.8 (26.2) | 0.001 | <0.001 | <0.001 |
|  | All | 328 | 6.3 (16.4) | 212 | 18.3 (20.4) | 80 | 38.1 (24.6) | <0.001 | <0.001 | <0.001 |
| Work productivity loss | 4-week | 110 | 5.6 (15.2) | 84 | 20.1 (21.6) | 34 | 40.8 (24.8) | <0.001 | <0.001 | <0.001 |
|  | 3-month | 112 | 9.6 (21.8) | 71 | 19.3 (24.3) | 24 | 41.1 (27.7) | 0.096 | <0.001 | 0.007 |
|  | 6-month | 106 | 6.2 (14.8) | 57 | 23.8 (22.5) | 21 | 46.1 (27.3) | <0.001 | <0.001 | <0.001 |
|  | All | 328 | 7.2 (17.7) | 212 | 20.8 (22.8) | 79 | 42.3 (26.1) | <0.001 | <0.001 | <0.001 |
| Activity impairment | 4-week | 160 | 8.2 (18.5) | 109 | 23.5 (22.2) | 59 | 48.6 (24.7) | <0.001 | <0.001 | <0.001 |
|  | 3-month | 155 | 10.3 (20.0) | 98 | 25.1 (25.7) | 39 | 42.3 (30.0) | 0.001 | <0.001 | 0.002 |
|  | 6-month | 147 | 7.1 (16.7) | 77 | 24.3 (22.7) | 36 | 53.3 (25.1) | <0.001 | <0.001 | <0.001 |
|  | All | 462 | 8.6 (18.5) | 284 | 24.3 (23.5) | 134 | 48.1 (26.6) | <0.001 | <0.001 | <0.001 |

Footnote:

*^a^* P value based on mixed models for repeated measurements. Models include time, category of number of symptoms and their interaction, controlling for pre-COVID-19 score, index vaccination status and its interaction with time, age, gender, race/ethnicity, region, social vulnerability index category, number of acute respiratory infection symptoms on index day, previously tested positive, high-risk settings, and immune-compromised conditions.

**Table S4**. *Proportions of Patient Clusters Over Time by BNT162b2 Vaccination Status*

| Vaccination status | Time | Cluster 1: < 2 symptoms | Cluster 2: 2 – 6 symptoms | Cluster 3: > 6 symptoms | P value ^a^ | |
| --- | --- | --- | --- | --- | --- | --- |
|  |  |  |  |  | vs. Week 4 | vs. Month 3 |
| Boosted | Week 4 | 48 (55.2%) | 34 (39.1%) | 5 (5.7%) | - | - |
|  | Month 3 | 47 (64.4%) | 24 (32.9%) | 2 (2.7%) | 0.119 | - |
|  | Month 6 | 54 (80.6%) | 11 (16.4%) | 2 (3.0%) | 0.001 | 0.038 |
| Primed | Week 4 | 40 (46.5%) | 32 (37.2%) | 14 (16.3%) | - | - |
|  | Month 3 | 39 (50.6%) | 28 (36.4%) | 10 (13.0%) | 0.343 | - |
|  | Month 6 | 37 (51.4%) | 23 (31.9%) | 12 (16.7%) | 0.491 | 0.899 |
| Unvaccinated | Week 4 | 72 (46.5%) | 43 (27.7%) | 40 (25.8%) | - | - |
|  | Month 3 | 69 (48.6%) | 46 (32.4%) | 27 (19.0%) | 0.144 | - |
|  | Month 6 | 56 (46.3%) | 43 (35.5%) | 22 (18.2%) | 0.298 | 0.832 |

^a^ *P* value for the differences between least-square estimates from the ordinal logistic model for number-based category. Model included time, index vaccination status and its interaction with time, controlling for age, gender, race/ethnicity, region, social vulnerability index category, number of acute respiratory infection symptoms on index day, previously tested positive, high-risk settings, and immune-compromised conditions.
