# Supplementary material for: Associations between symptom-based long COVID clusters and long-term quality of life, work and daily activities among individuals testing positive for SARS-CoV-2 at a national retail pharmacy
Source: J Patient Rep Outcomes. 2024 Oct 22;8:122. doi: 10.1186/s41687-024-00797-7 (PMC11496399; doi:10.1186/s41687-024-00797-7)
Supplement: Supplementary file 2 — Supplementary Material 2 [file 41687_2024_797_MOESM2_ESM.docx]

**Figure S1. Patient Flow**


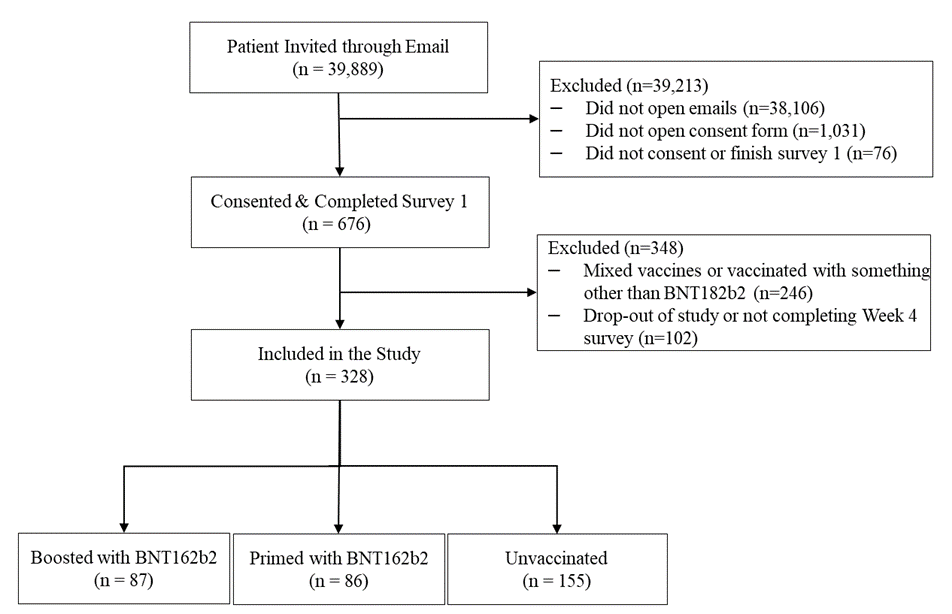


**Figure S2**. *Symptom Burden and Working Hours by Cluster and Time Point*

**
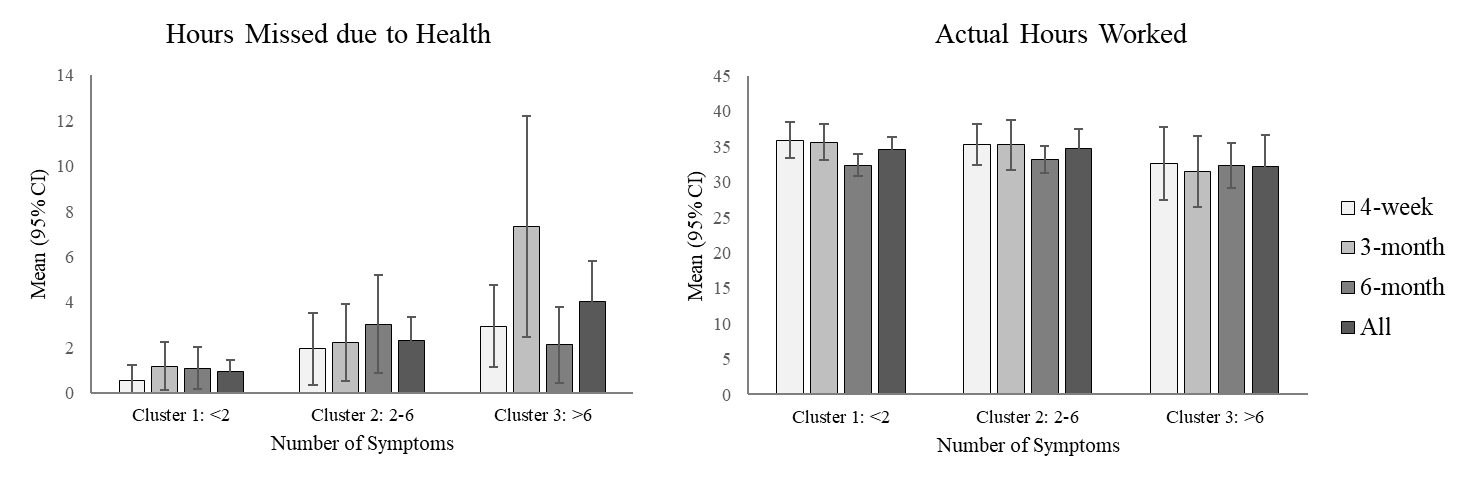
**

A) Hours missed to health considerations and B) actual hours worked reported by COVID-19-related symptoms (low, < 2; moderate, 2 – 6; high, > 6) across time points (4-week, blue; 3-month, orange; 6-month, grey; all, yellow) reported as the mean with 95% confidence interval.
